# A P2X receptor-mediated nociceptive afferent pathway to lamina I of the spinal cord

**DOI:** 10.1186/1744-8069-1-4

**Published:** 2005-01-17

**Authors:** Meng Chen, Jianguo G Gu

**Affiliations:** 1 Department of Oral and Maxillofacial Surgery, McKnight Brain Institute and College of Dentistry, University of Florida, Gainesville, Florida, 32610, USA

## Abstract

Of the six lamina regions in the dorsal horn of the spinal cord, lamina I is a major sensory region involved in nociceptive transmission under both physiological and pathological conditions. While P2X receptors have been shown to be involved in nociception, it remains unknown if P2X receptors are involved in nociceptive transmission to lamina I neurons. Using rat spinal cord slice preparations and patch-clamp recordings, we have demonstrated that the excitatory synaptic transmission between primary afferent fibers and lamina I neurons is significantly affected by ATP and α,β-methylene-ATP. The synaptic effects of them include the increases of the frequency of both miniature excitatory postsynaptic currents (mEPSCs) and spontaneous EPSCs (sEPSCs), and decreases of evoked EPSCs (eEPSCs). These effects were blocked by pyridoxalphosphate-6-azophenyl-2', 4'-disulfonic acid (PPADS, 10 μM) and suramin (30 μM). In the neurons for which ATP and α,β-methylene-ATP had effects on mEPSCs, sEPSCs and eEPSCs, capsaicin produced similar synaptic effects. Our results indicate that P2X receptors are expressed on many afferent fibers that directly synapse to lamina I neurons. Furthermore, these P2X receptor-expressing afferent fibers are capsaicin-sensitive nociceptive afferents. Thus, this study reveals a P2X receptor-mediated nociceptive afferent pathway to lamina I of the spinal cord and provides a new insight into the nociceptive functions of P2X receptors.

## Background

Spinal cord dorsal horn, the first central site for sensory processing, is divided into structurally and functionally distinct lamina regions [1]. Lamina I, or marginal zone of the spinal cord dorsal horn, is a critical region in nociceptive transmission. Different from other lamina regions, many lamina I neurons receive nociceptive inputs and directly relay the nociceptive information through ascending pathways to the brain [1,2]. Nociceptive transmission through lamina I and their modulation there have important implications in both physiological and pathological pain conditions [2,3]. Thus, a chemical mediator may have significant influence on pain conditions if it has an effect on this nociceptive pathway.

Extracellular ATP is a chemical mediator that has multiple effects in different tissues including nervous systems [4]. ATP is involved in sensory signaling at peripheral sites [5-7] and synaptic modulation at central sites in the somatic sensory system [8,9]. At the periphery, ATP may directly stimulate nociceptive afferent fibers through the activation of P2 receptors, resulting in nociceptive inputs to the dorsal horn of the spinal cord or the equivalent sensory structure in the brain [6,10]. At the central sites in the dorsal horn of the spinal cord, the inner part of lamina II (lamina IIi) may be a region where ATP-induced sensory inputs are transmitted. This is based on the strong P2X3 receptor-immunoreactivity in this region [11] as well as previous electrophysiological evidence [12,13]. Lamina V may be another CNS site where ATP-sensitive inputs are transmitted. Many neurons in lamina V region receive sensory inputs with a wide dynamic range including both nociceptive and non-nociception signals [1]. Previous studies have shown that ATP modulates synaptic transmission to lamina V of the dorsal horn. The synaptic modulation is mediated by the activation of P2X receptors and these P2X receptors are localized at the presynaptic terminals of afferent fibers innervating lamina V neurons. The ATP-sensitive afferent terminals to lamina V neurons were found to be capsaicin-insensitive [14].

ATP and its receptors (P2 receptors) have been shown to be involved in inflammatory and neuropathic pain conditions [15,16]. P2 receptors may be therapeutic targets for the management of pathological pain conditions [17,18]. Although the significance of lamina I neurons in inflammatory and neuropathic pain conditions have been well recognized [19,20], it is currently not known whether lamina I is one of the CNS sites for transmitting ATP-sensitive nociceptive inputs. This issue is important for understanding how ATP and its receptors are involved in nociceptive transmission under physiological and pathological conditions. This critical information has not been provided in the previous studies, which is mainly due to the difficulty in making electrophysiological recordings from lamina I neurons [2,3]. In the present study, we performed patch-clamp recordings from lamina I neurons and studied effects of ATP and its analog αβmeATP on excitatory synaptic transmission to lamina I neurons. We demonstrated, for the first time, a P2X receptor-mediated nociceptive pathway to the lamina I of the spinal cord dorsal horn.

## Results

### Effects of ATP and αβmeATP on miniature EPSCs of lamina I neurons

**Figure 1:**
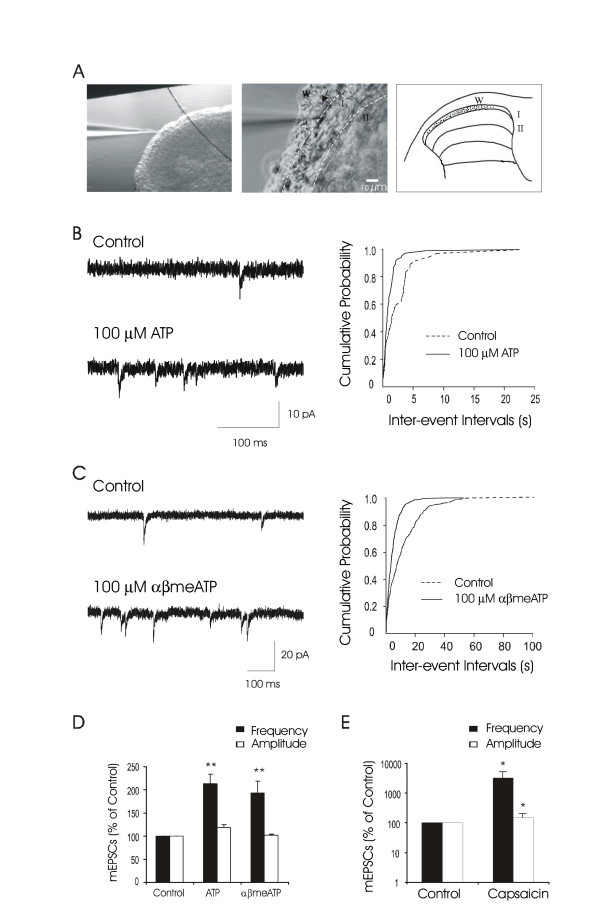
**Effects of ATP and αβmeATP on mEPSCs recorded from lamina I neurons of rat spinal cord slice preparations A. **The image on the left shows a spinal cord slice section viewed under a 4X objective. An electrode is shown to the left side of the tissue section. The tip of the electrode is in lamina I. Scale bar: 100 μm. The image in the middle shows the tissue section viewed under a 40X objective with the IR-DIC system. Near the center of the field is a lamina I neuron (arrow indicated) that is patched with an electrode. The cell is on the border between white matter and gray matter. White matter (W), lamina I (I), and lamina II (II) are indicated. Lamina I region is outlined with two dash lines. Scale bar: 10 μm. The drawing on the right indicates the locations of all neurons recorded in this study. **B. **Representative traces show mEPSCs at the basal level (control) and following the application of 100 μM ATP. The graph on the right shows cumulative probability histogram of inter-event intervals in the same neuron. The inter-event intervals, which reflect the changes of mEPSC frequency, were significantly shifted following the application of 100 μM ATP (P < 0.01, Kolmogorov-Smirnov test). **C. **The experiment was the same as **B **except 100 αβmeATP was tested. The inter-event intervals were significantly shifted following the application of 100 μM αβmeATP (P < 0.01, Kolmogorov-Smirnov test). **D. **The bar graph shows pooled results from experiments represented in **B **and **C. **Both ATP (n = 13) and αβmeATP (n = 14) increased mEPSC frequency without affecting mEPSC amplitude. **E. **Effects of capsaicin (2 μM) on mEPSCs in 8 cells that were tested for ATP and αβmeATP in D. The scale is in logarithm. Data represent Mean ± SEM, *p < 0.05, **p < 0.01, compared with controls, paired Student's *t*-test.

We performed patch-clamp recordings from lamina I neurons in spinal cord slice preparations (Figure 1A). Under 40X objective with IR-DIC microscopic system, white matter and gray matter of the dorsal horn could be distinguished. All recordings (Figure 1A) were made from dorsal horn neurons whose somas were either within or adjacent to the white matter (within a distance of 30 μm).

We first studied whether lamina I neurons directly received synaptic inputs from ATP-sensitive presynaptic terminals. This was done by testing the effects of ATP (100 μM) on mEPSCs recorded from lamina I neurons. Miniature EPSCs were recorded in the presence of 10 mM lidocaine. The use of the high concentration of lidocaine was to block both TTX-sensitive and TTX-resistant Na^+ ^channels so that the effects of ATP reflected its direct action at the synaptic sites of the recorded neurons [21]. In this study, basal levels of mEPSCs were first recorded for 10 min. Miniature EPSC frequency and mEPSC amplitude at basal levels were served as controls, which had a variation within ± 10% in the same cells. We defined an increase of mEPSC frequency and amplitude to be more than 120% of control following ATP application, provided they returned to basal levels after washout of ATP. Bath application of 100 μM ATP for 2 min increased mEPSC frequency in 13 out of 30 lamina I neurons recorded (Figure 1B,D). Miniature EPSC frequency of these 13 cells increased to 210 ± 22% of control, from 0.56 ± 0.11 Hz at the basal level to 1.08 ± 0.19 Hz following the application of 100 μM ATP (n = 13, P < 0.01, Figure 1D). Miniature EPSC frequency in all these 13 cells returned to basal levels 10 min after washout of ATP. In the cells for which mEPSC frequency was increased, the amplitude of mEPSCs was not significantly changed following ATP application (Figure 1D; 118 ± 7% control, 11 ± 1 pA in control and 12.0 ± 0.9 pA following ATP application, n = 13, P > 0.05). For the remaining 17 cells, neither mEPSC frequency nor mEPSC amplitude was increased following ATP application (not shown). In all the cells recorded, we did not observe any direct whole-cell inward current during ATP application, a result consistent with previous studies in tissue slice preparations. In the above experiments and the experiments described below, 100 μM ATP was applied in the presence of 10 μM ARL67156, an ecto-ATPase inhibitor that was used to prevent ATP metabolism [14].

We tested αβmeATP, a metabolically stable ATP analog, on mEPSCs recorded from lamina I neurons. Similar to ATP, application of 100 μM αβmeATP increased mEPSC frequency in 14 out of 35 lamina I neurons (Figure 1C,D). Of these 14 cells, mEPSC frequency increased to 195 ± 25% of control (n = 14, P < 0.01, Figure 1D). Miniature EPSC amplitude in these cells was not significantly different from the control (Figure 1D; 102 ± 2% control, n = 14, P > 0.05). In the above experiments, αβmeATP did not directly evoke whole-cell inward currents in any cell. These results provided electrophysiological evidence indicating that the presynaptic terminals contacting the recorded lamina I neurons are ATP-sensitive/αβmeATP-sensitive. The expression of P2 receptors at these presynaptic terminals is suggested.

To test whether ATP-sensitive presynaptic terminals that contacted lamina I neurons were potentially related to nociceptive transmission, we tested capsaicin sensitivity in 8 cells that had responses to αβmeATP and ATP. Capsaicin was used because it selectively actives a subset of nociceptive afferent fibers that express VR1 receptors. We found that all of the 8 αβmeATP-responsive neurons also responded to capsaicin (2 μM) with a large increase of mEPSC frequency (Figure 1E; 0.4 ± 0.1 Hz in control *vs *18 ± 7 Hz following capsaicin, n = 8, P < 0.05). Miniature EPSC amplitude was also found to be increased by capsaicin (10.8 ± 1.5 pA in control *vs *15.8 ± 2.2 following capsaicin, n = 8, P < 0.05), which was most likely due to the high mEPSC frequency that produced temporal peak summation. There was no direct whole-cell inward current that was observed during capsaicin application.

### Effects of ATP and its analogs on spontaneous EPSCs recorded from lamina I neurons

**Figure 2:**
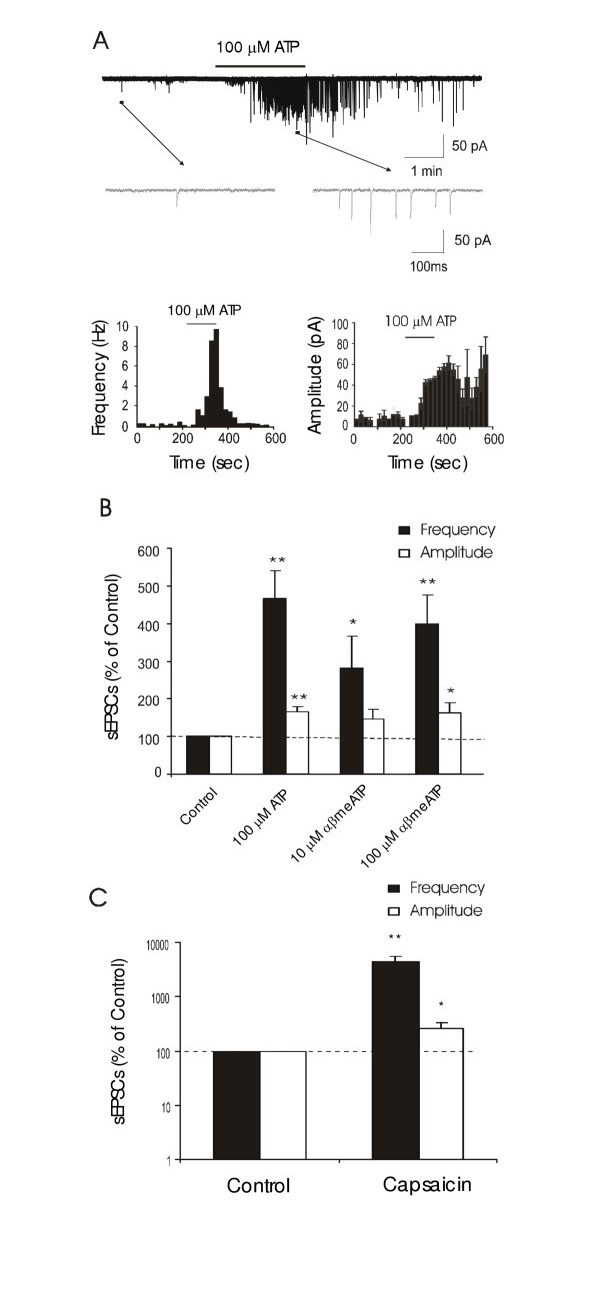
**Effects of ATP and αβmeATP on sEPSCs of lamina I neurons A. **The trace on the tope panel shows sEPSCs recorded from a lamina I neuron before and following the application of 100 μM ATP. Two small portions, one before ATP and the other following ATP application (arrows indicated), are shown at the expanded scale. Two histograms below the traces show time courses of sEPSC frequency (left) and amplitude (right) in the same cell. Time bin is 20 sec. **B. **The bar graph shows pooled results of the effects of ATP (100 μM, n = 44), 10 μM αβmeATP (n = 8), and 100 μM αβmeATP (n = 26) on sEPSC frequency (filled bars) and amplitude (open bars). Data were normalized by the basal levels. **C. **The bar graph shows effects of capsaicin on sEPSCs in 6 neurons that were tested in **B **with 100 μM ATP and 100 μM αβmeATP.

We tested effects of P2 receptor agonists on spontaneous EPSCs (sEPSCs). In these experiments, Na^+ ^channels were not blocked so that action potentials were permitted to be generated. This allowed us to observe a more global effect of P2 receptor agonists on synaptic activity in lamina I. Of 106 lamina I neurons that were recorded, 44 cells showed increases in sEPSC frequency after application of 100 μM ATP (Figure 2A,B). The changes of sEPSC frequency in these 44 cells were 474 ± 70% of control (Figure 2B; 0.50 ± 0.09 Hz in control *vs *1.98 ± 0.30 Hz following ATP, n = 44, P < 0.01). sEPSC amplitude was also significantly increased (Figure 2B; 157 ± 14% of control, 13.4 ± 1.0 pA in control *vs *19.8 ± 2.3 pA following ATP, n = 44, P < 0.01,).

Similar response was observed when αβmeATP was tested. Of 47 cells tested with 100 μM αβmeATP, 26 of them showed increases in sEPSC frequency and amplitude. The change of sEPSC frequency was 358 ± 67% of control (Figure 2B, n = 26, P < 0.01,). The change of sEPSC amplitude was 163 ± 23% of control (Figure 2B, n = 26, P < 0.05). At a lower concentration of 10 μM αβmeATP, 8 out of 19 cells showed increases of sEPSC frequency and the change of sEPSC frequency in these cells was 224 ± 62% of control (Figure 2B, n = 8, P < 0.05).

We further tested effects of capsaicin on sEPSCs in 6 cells that showed the increases of sEPSC by αβmeATP and ATP. All 6 cells showed significant increases of sEPSC frequency following the application of 2 μM capsaicin (Figure 2C; 0.55 ± 0.18 Hz in control *vs *22.1 ± 7.8 Hz following capsaicin, n = 6). sEPSC amplitude was also found to be significantly increased by 2 μM capsaicin (Figure 2C; 14.6 ± 4.9 pA in control *vs *22.5 ± 3.5 pA following capsaicin, n = 6).

### Effects of P2 antagonists on ATP-induced increases of sEPSCs

**Figure 3:**
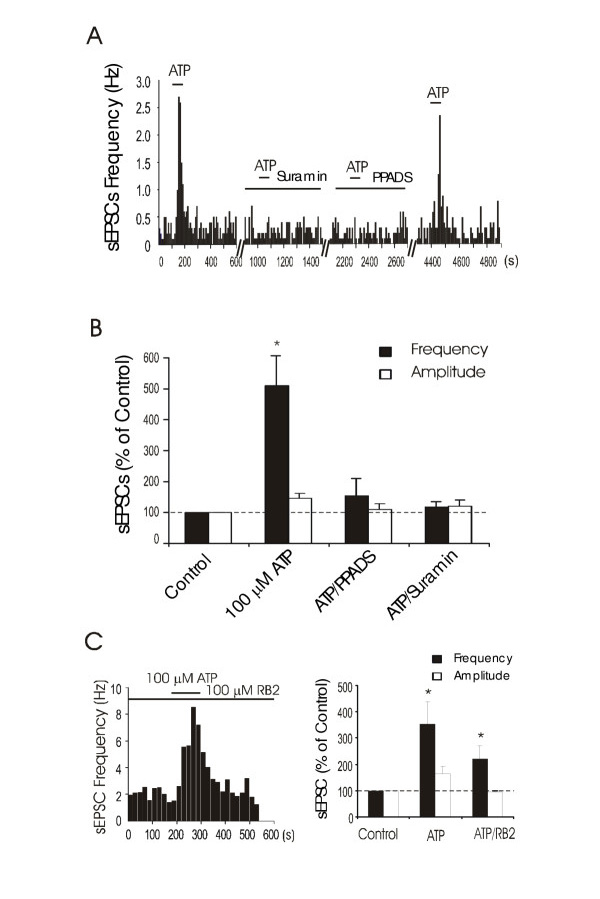
**Block of ATP-induced synaptic responses by P2 antagonists A. **The histogram shows the effects of ATP on sEPSC frequency in a lamina I neuron in the absence and presence of suramin (30 μM) or PPADS (10 μM). Suramin and PPADS were pre-applied for 10 min and were present during the recording. After 30 min washout of antagonists, 100 μM ATP was applied again for a test of recovery. **B. **Summarized data show effects of ATP on sEPSC frequency (filled bars) and amplitude (open bars) in the absence (n = 5) and presence of PPADS (10 μM, n = 4) and suramin (30 μM, n = 3). **C. **Histogram on the left shows a time course of ATP-induced increase of sEPSC frequency in a lamina I neuron in the presence of 100 μM reactive blue 2(RB2). Bar graph on the right is a summary that shows the effects of 100 μM ATP on the sEPSCs in the absence and presence of 100 μM RB2 (n = 11).

We tested the effects of PPADS and suramin on ATP-induced increases of sEPSCs (Figure 3A,B). Of 5 cells for which 100 μM ATP increased sEPSC frequency to 512 ± 127% of basal levels in the absence of PPADS, sEPSC frequency was 155 ± 52% of the basal levels following 100 μM ATP in the presence of 10 μM PPADS (n = 4), and was 117 ± 17% of basal levels following 100 μM ATP in the presence of 30 μM suramin (n = 3). Similarly, αβmeATP-induced increases of sEPSCs in 3 cells (294 ± 78% of basal sEPSC frequency) were abolished in the presence of 10 μM PPADS (117 ± 14% of basal sEPSC frequency). These results suggested that P2 receptors are involved in the increases of spontaneous excitatory synaptic activity to lamina I neurons.

While ATP-induced increases of sEPSCs were abolished by both PPADS and suramin, ATP still could increase sEPSCs in the presence of reactive blue 2, a P2Y receptor antagonist. As shown in Figure 3C, in 11 cells showing increases of sEPSCs in response to 100 μM ATP (354 ± 85% of basal levels, P < 0.05), 100 μM ATP increased the sEPSC frequency to 221 ± 49% of basal levels when 100 μM RB-2 was present (P < 0.05, compared with basal levels, Figure 3C). Taken together, these results suggest that P2X receptors are involved in ATP-induced increases of sEPSCs.

### Effects of ATP and αβmeATP on synaptic inputs from primary afferent fibers to lamina I neurons

**Figure 4:**
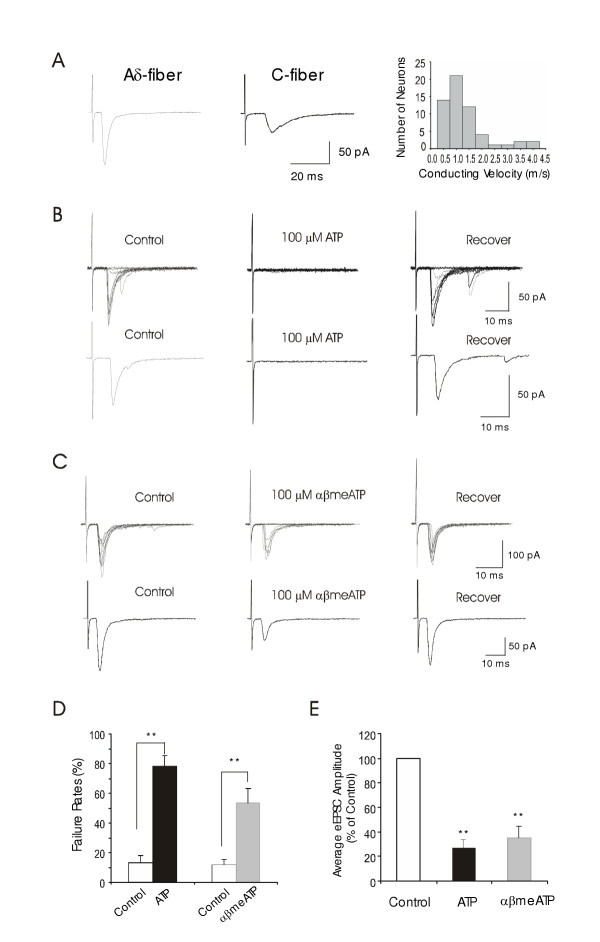
**Suppression of eEPSCs by ATP and αβmeATP A. **The trace on the left shows an averaged eEPSC recorded from a lamina I neuron that received monosynaptic inputs from Aδ-afferent fibers. The averaged eEPSC was obtained from eEPSCs elicited by 10 sweeps of stimuli. The trace on the right is an averaged eEPSC from a lamina I neuron that received monosynaptic inputs from C-fibers. The graph shows numbers of lamina I neurons recorded for all the eEPSC experiments in this study and their corresponding afferent conduction velocity (n = 57). **B. **Top panel shows superimposed eEPSCs following 10 sweeps of stimuli in normal bath solution (control), following the application of 100 μM ATP, and washout of ATP (recovery). The averaged eEPSCs from 10 sweeps of stimuli are shown in the bottom panel. **C. **Similar to **B **except αβmeATP was tested in a different lamina I neuron. **D. **The bar graph summarizes the increases of synaptic failure rates by 100 μM ATP (black bar, n = 9) and 100 μM αβmATP (gray bar, n = 18). **E. **A summary of the reduction of average eEPSC amplitude by 100 μM ATP (black bar, n = 9) and 100 μM αβmATP (gray bar, n = 18).

To further demonstrate that ATP-sensitive terminals are derived from primary afferent fibers, we studied effects of ATP and αβmeATP on synaptic inputs elicited by stimulation of dorsal root (i.e. primary afferent fibers). Dorsal root stimuli resulted in the evoked EPSCs (eEPSCs) recorded from lamina I neurons. Monosynaptic eEPSCs showed large variations in their latency among different cells (Figure 4A). The conduction velocity of afferent inputs, calculated from the latency of eEPSCs and the length of the dorsal roots, was from 0.33 to 4 m/s (1.1 ± 0.11 m/s, n = 57, Figure 4A), in the range of C-fiber and Aδ-fiber conduction velocity at these ages of rats [22]. Synaptic failure rates were low before the application of ATP and αβmeATP. However, following the application of 100 μM ATP or 100 μM αβmeATP, there was a significant increase in synaptic failure rates and a significant decrease of the averaged eEPSC amplitude in many lamina I neurons (Figure 4B to 4E). Of 13 cells tested with 100 μM ATP in the presence of 10 μM ARL67156 and 2 mM caffeine, 9 of them showed increases in synaptic failure rates and decreases in the averaged eEPSC amplitude (Figure 4B,D). The failure rates were 8 ± 3% in control and increased to 78 ± 7% following 100 μM ATP applications (n = 9, P < 0.01); the averaged eEPSC amplitude was 35 ± 9 pA in control and reduced to 12 ± 4 pA following 100 μM ATP application (24 ± 7% of control, n = 9, P < 0.01). Of 29 cells tested with 100 μM αβmeATP, 18 of them showed depression of the averaged amplitude of eEPSCs, accompanied with the increases of failure rates (Figure 4C,E). The failure rates were 12 ± 5% in control and increased to 53 ± 10% following 100 μM αβmeATP applications (n = 18); the eEPSC amplitude was reduced to 35 ± 7% of control following 100 μM αβmeATP application (n = 18, p < 0.01).

**Figure 5:**
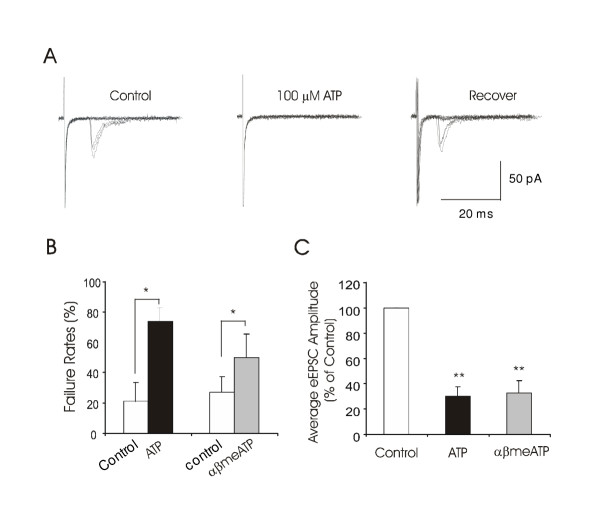
**Suppression of eEPSCs by ATP and αβmeATP in the presence of bicuculline A. **The traces show eEPSCs recorded from a lamina I neurons under the following conditions: in bath solution with 20 μM bicuculline (control), following the application of 100 μM ATP in the presence of 20 μM bicuculline, and washout of ATP (recovery). In each set of a test, eEPSCs were elicited by 10 sweeps of stimuli. **B. **The bar graph shows a pooled result of increases in synaptic failures by ATP (100 μM, n = 5) and αβmeATP (100 μM, n = 6). **C. **A summary shows the decreases of average eEPSCs by 100 μM ATP (n = 5) and 100 μM αβmeATP (n = 6). All recordings were made from lamina I neurons in the presence of 20 μM bicuculline.

The suppression of eEPSCs could be due to a direct action on primary afferent axons whose terminals synapse to lamina I neurons or due to an effect indirectly through GABAergic presynaptic inhibition. To exclude the latter possibility, we tested the effects of 100 μM ATP and 100 μM αβmeATP on eEPSCs in the presence of the GABA-A receptor inhibitor bicuculline (20 μM). We found that both ATP and αβmeATP still could suppress eEPSCs (Figure 5). Of 7 cells tested with 100 μM ATP in the presence of bicuculline, 5 of them showed suppression of eEPSCs. Synaptic failure rates increased from 26 ± 6% before ATP to 68 ± 15% following 100 μM ATP (n = 5, p < 0.01, Figure 5B). The averaged eEPSC amplitude was also significantly decreased (Figure 5C). Of 14 cells tested with 100 μM αβmeATP in the presence of bicuculline, 6 of them showed depression of eEPSCs. For these 6 cells, synaptic failure rates were increased from 27 ± 10% before αβmeATP to 50 ± 15% following the application of 100 μM αβmeATP (n = 6, p < 0.05, Figure 5B), the averaged eEPSC amplitude decreased to 33% ± 9% of control following 100 μM αβmeATP (n = 6, p < 0.01, Figure 5C). These results suggest that the suppression of primary afferent synaptic inputs to lamina I neurons was due to a direct action of ATP or αβmeATP on primary afferent fibers.

**Figure 6:**
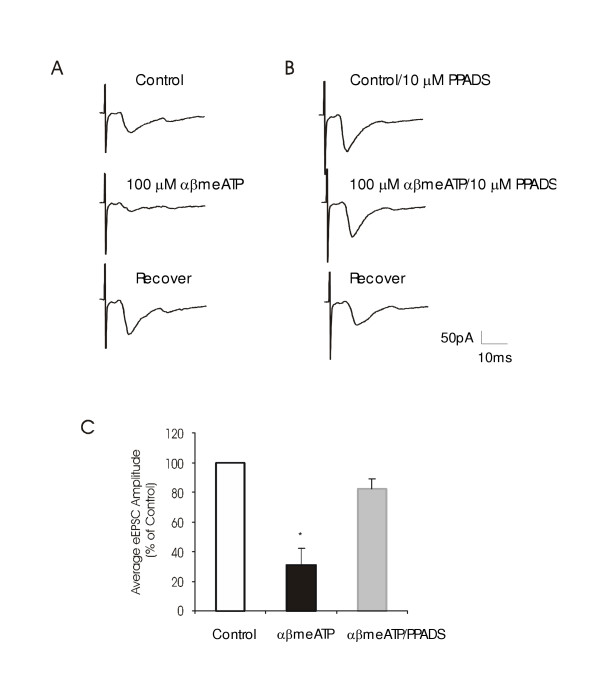
**Block of αβmeATP-induced suppression of eEPSCs by PPADS A. **The three traces on the top, middle and bottom panels show eEPSCs recorded from a lamina I neuron in normal bath solution (Control), during the application of 100 μM αβmeATP and after washout of αβmeATP (Recover), respectively. **B. **The same neuron shown **A **was tested again in the presence of 10 μM PPADS. In both **A **and **B**, a trace of eEPSC was obtained from an average of eEPSCs evoked by 10 sweeps of stimuli. **C. **Pooled results (n = 4) show effects of 100 μM αβmeATP on eEPSC amplitude in the absence and presence of 10 μM PPADS. Data represent percent of control values, and eEPSCs in control is scaled as 100%.

If P2X receptors were involved in the suppression of eEPSCs, the suppression should be abolished in the presence of P2X antagonists. We tested the effects of PPADS on αβmeATP-induced suppression of eEPSCs (Figure 6). Of 4 cells for which 100 μM αβmeATP suppressed eEPSC amplitude to 31 ± 11% of control (n = 4, P < 0.05) in the absence of PPADS, αβmeATP did not significantly suppress eEPSC amplitude when 10 μM PPADS was present (82 ± 7% of control, n = 4, Figure 6C). Thus, P2X receptors are expressed on primary afferent fibers that contact lamina I neurons.

**Figure 7:**
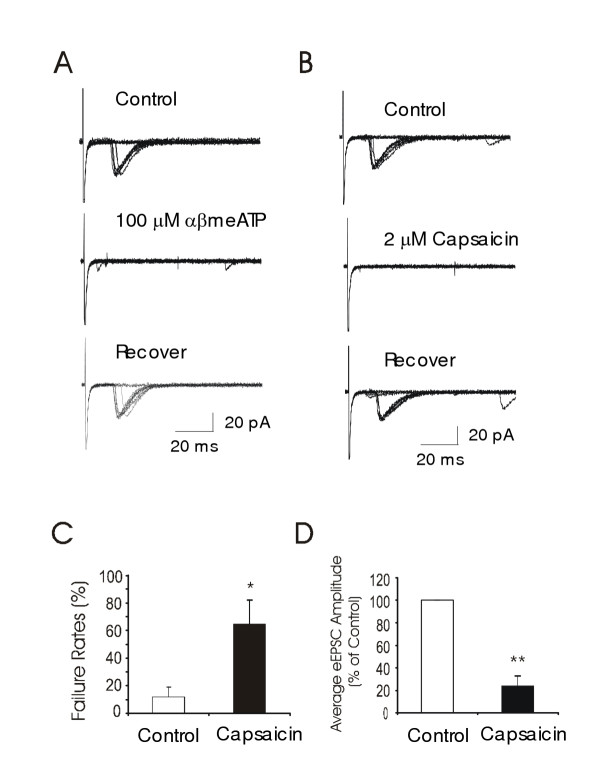
**Capsaicin-sensitivity of αβmeATP-sensitive primary afferents to lamina I A. **Example recordings from a lamina I neuron show eEPSCs following 10 sweeps of stimuli in normal bath solution (control), following the application of 100 μM αβmeATP, and washout of the drug (recovery). **B. **Capsaicin (2 μM) was tested in the same neuron as shown in **A**. **C. **The bar graph summarizes capsaicin-induced synaptic failures in 6 lamina I neurons that received ATP/αβmeATP-sensitive primary afferent inputs. **D. **A summary shows capsaicin-induced reduction of averaged eEPSC amplitude (n = 6). All neurons were recorded from lamina I that received ATP/αβmeATP-sensitive primary afferent inputs.

To study whether ATP-sensitive afferent fibers that synapsed to lamina I neurons were nociceptive afferent fibers, we tested capsaicin sensitivity of the evoked eEPSCs. Of the 6 lamina I neurons that showed eEPSC suppression by ATP and αβmeATP (Figure 7A), all of them also showed eEPSC suppression following the application of 2 μM capsaicin (Figure 7B,C, D). The synaptic failure rates of these 6 cells were 12 ± 6% before capsaicin and increased to 65 ± 16% (n = 6, p < 0.05) following the application of 2 μM capsaicin. The averaged amplitude of eEPSCs was 46 ± 17 pA before capsaicin and reduced to 18 ± 10 pA following 2 μM capsaicin (n = 6, p < 0.01). Of these six recordings, the afferent conduction velocities were less than 1 m/s (from 0.5 to 0.8 m/s) in 4 cells and more than 1 m/s (1.1 and 1.5 m/s) in 2 cells. These results suggest that ATP-sensitive afferent fibers that synapse to lamina I neurons are nociceptive afferent fibers.

## Discussion

In the present study we have shown that ATP and its analog αβmeATP produce profound effects on excitatory synaptic transmission to lamina I neurons of the spinal cord dorsal horn. Pharmacological tests indicate that P2X receptors are involved in the synaptic responses and that P2X receptors are expressed on many capsaicin-sensitive afferent fibers innervating lamina I neurons. In view of the significance of lamina I in transmitting nociceptive signals and in the development of pathological pain conditions [2,3], our findings provide a new insight into P2X receptor functions in physiological and pathological pain conditions [16,17,23].

In this study, the presynaptic actions of ATP and αβmeATP were evidenced electrophysiologically by their effects on mEPSCs and eEPSCs [21]. Both ATP and αβmeATP increased mEPSC frequency without affecting mEPSC amplitude; both ATP and αβmeATP also increased synaptic failure rates of eEPSCs. The involvement of P2X receptors is supported by several lines of pharmacological evidence. First, αβmeATP is a selective agonist to P2X receptors [24] and its effects on mEPSCs, sEPSCs and eEPSCs were similar to ATP in this study. Second, at low micromolar concentrations, PPADS is a selective antagonist for P2X receptors [24]; we found that PPADS at 10 μM abolished synaptic effects of ATP and αβmeATP. Third, ATP and αβmeATP still could induce synaptic responses in the presence of the P2Y receptor antagonist RB2. Fourth, the potential complication by the ATP metabolite adenosine was excluded with the use of caffeine to block adenosine receptors and the used of ARL67156 to prevent ATP metabolism [14].

We have found that in the same lamina I neurons for which ATP and αβmeATP had synaptic effects, capsaicin produced similar responses. For example, in the lamina I cells for which αβmeATP increased synaptic failure rates of eEPSCs, capsaicin also enhanced synaptic failure rates in these cells. These findings suggest that many ATP- and αβmeATP-sensitive presynaptic terminals to lamina I neurons were derived from capsaicin-sensitive nociceptive afferent fibers. Our previous study showed that P2X receptor-expressing afferent fibers innervating lamina V neurons were capsaicin-insensitive Aδ-fibers [14]. In the present study, we have found that P2X receptor-expressing afferent fibers to lamina I are capsaicin-sensitive and could be either Aδ or C-fibers. Thus, the P2X-expressing afferent fibers are distinct in their lamina distribution and in their capsaicin sensitivity. The lamina I innervation and the capsaicin sensitivity of these ATP-sensitive afferent fibers strongly suggest the nociceptive function of these P2X receptors.

The synaptic failures of eEPSCs by ATP and αβmeATP could be due to conduction block following the activation of P2X receptors on primary afferent axons. Alternatively, the increase of synaptic failure rates was caused by presynaptic inhibition through GABAergic inhibitory interneurons [25]. This latter possibility, however, is discounted because ATP and αβmeATP still increased the failure rates of the evoked EPSCs in the presence of bicuculline to block GABA-A receptors. These results further support the idea that P2X receptors are expressed on primary afferent fibers innervating lamina I neurons. The synaptic failure of eEPSCs following P2X receptor activation does not discount the involvement of P2X receptors in transmitting nociceptive signals to lamina I regions. In opposite, it suggests that P2X receptor action may directly depolarize primary afferent fibers to initiate nociceptive signals, which are then transmitted to lamina neurons. Consistent with this idea, out results showed that both frequency and amplitude of sEPSCs were significantly increased following the application of ATP or αβmeATP.

Of more than 10 subtypes of functional P2X receptors identified so far, low concentrations of αβmeATP selectively activate those that contain P2X1 or P2X3 subunits [24]. Thus, the sensitivity to 10 μM αβmeATP in our study narrows down the possible P2X receptor subtypes [26]. The sensitivity to the block by 10 μM PPADS and 30 μM suramin in our study may allow us to further exclude a number of P2 receptors including P2Y receptors, homomeric P2X4 receptors, homomeric P2X6 receptors, and homomeric P2X7 receptors [26]. Previous studies have shown the involvement of P2X3 receptors in nociception [7]. However, because P2X3-expressing afferent terminals are restricted in the inner layer of lamina II [11], it is less likely that the functional P2X receptors in our study are P2X3-containing subtypes. The P2X receptor subtype(s) involved in the nociceptive pathway shown in this study may not be easily identified due to the lack of selective agonists and antagonists for most of P2X receptor subtypes and due to the possible presence of more P2X receptor subtypes. The answer to this critical issue in future studies may provide new therapeutic targets for pain management.

## Methods

### Spinal cord slice preparation

Principles of laboratory animal care (NIH publication No. 86-23, revised 1985) were followed in all the experiments described in the present study. Spinal cord slice preparations and patch-clamp recordings were described in details in our previous studies [14]. In brief, Sprague Dawley rats at the postnatal age of 10–21 days were used. Transverse spinal cord slices were prepared from lumbar enlargement of the spinal cords. Two types of spinal cord slice sections were made. One type was in the thickness of 400 μm without dorsal root, and was used for studying mEPSCs and sEPSCs. The other type was in the thickness of 600 μm with attached dorsal roots, and was use for experiments testing eEPSCs. The length of the dorsal roots was in a range of 8–12 mm. The spinal cord slices were maintained in a basket submerged in ~200 ml Krebs solution (24°C). The Krebs solution contained (in mM): NaCl 117, KCl 3.6, CaCl_2 _2.5, MgCl_2 _1.2, NaH_2_PO_4 _1.2, NaHCO_3 _25 and glucose 11; the solution was saturated with 95 % O_2 _and 5% CO_2 _and had pH of 7.3.

### Patch-clamp recordings

In each experiment a spinal cord slice was transferred to a 0.5 ml recording chamber and placed on the stage of an upright microscope. The microscope was equipped with an infrared differential interference contrast (IR-DIC) system. The spinal cord slice was superfused with the Krebs' solution flowing at 10 ml/min at room temperature (24°C); the Krebs' solution was equilibrated with 95% O_2 _and 5% CO_2_. Lamina I, the marginal region of the dorsal horn, was identified under the microscope. Individual neurons were identified with a 40× water immersion objective. Whole-cell patch-clamp recordings were made from lamina I neurons with electrodes filled with an internal solution containing (in mM): 135K-gluconate, 5KCl, 0.5CaCl_2_, 2MgCl_2_, 5EGTA, and 5HEPES; the pH of the solution was adjusted to 7.3 with NaOH. The resistances of the electrodes were ~5 MΩ when filled with the internal solution. The access resistance was below 30 MΩ and was not compensated. Signals were amplified and filtered at 2 kHz (Axopatch 200B) and sampled at 5 kHz using pCLAMP 7.0 (Axon instruments). EPSCs were recorded with cells held at -60 mV. The holding potential was close to the reversal potential for GABA_A _and glycine receptors under the experimental conditions so that the outward IPSCs were minimized and usually undetectable.

Spontaneous EPSCs were recorded in the normal Krebs' bath solution, and mEPSCs were recorded in the presence of 10 mM lidocaine. To record eEPSCs, stimuli were applied to a dorsal root with a suction electrode. Stimulation intensities were 60–150 μA for Aδ fiber and 200–800 μA for C-fibers; stimulation duration was always 0.1 msec. Evoked EPSCs were usually elicited by 10 sweeps of stimuli at a time interval of 5 s, which were used to obtain the average of eEPSCs. Monosynaptic eEPSCs were judged by a constant latency to the repeated stimuli. Conduction velocity was calculated from the latency of eEPSCs and the length of dorsal roots.

Effects of P2 agonists and capsaicin on mEPSCs, sEPSCs and eEPSCs were tested using ATP (100 μM), α,β-methylene-ATP (αβmeATP, 10 and 100 μM), and 2 μM capsaicin. When ATP was tested, bath solution always contained the ecto-ATP inhibitor ARL67156 (10 μM), and the adenosine receptor antagonist caffeine (2 mM) was also present. P2 antagonists including pyridoxalphosphate-6-azophenyl-2',4'-disulfonic acid (PPADS, 10 μM), suramin (30 μM) and reactive blue 2 (RB2, 100 μM) were used in this study. When the antagonists were tested, they were first pre-applied for 10 min, and then were co-applied with P2 agonists. The time intervals for multiple applications of testing compounds were 20 min. All compounds were applied via the bath solution. All compounds were purchased from Sigma (St. Louis, MO).

### Data analysis

Synaptic events including sEPSCs and mEPSCs were analyzed using Mini Analysis Program (Jaejin software, Anderson Place, GA) with criteria being the same as previously described [21,14]. In analyzing the changes of mEPSC frequency or sEPSC frequency following the bath application of P2 agonists, frequency time courses before and after P2 agonists were first constructed with time bin of 10 s. Then the average response in continuous 6 bins (60 s) around the peak was used to calculate the changes in reference to the basal level (control). Cells were assigned to be responsive to a test compound when there were more than 20% increases in mEPSC frequency or sEPSC frequency as previously described [21]. For eEPSCs, eEPSCs were elicited by 10 sweeps of stimuli, which were used for calculating the averaged amplitude of eEPSC. Cells were considered to be responsive to a testing compound when there were more than 20% increases in the averaged amplitude of eEPSCs. Unless otherwise indicated, data were presented as mean ± SEM. Paired Student's *t*-tests were used for statistical comparison, and significance was considered at the level of the p < 0.05.

## Competing interests

The author(s) declare that they have no competing interests.
